# Comutations in DDR Pathways Predict Atezolizumab Response in Non-Small Cell Lung Cancer Patients

**DOI:** 10.3389/fimmu.2021.708558

**Published:** 2021-09-24

**Authors:** Anning Xiong, Wei Nie, Yan Zhou, Changhui Li, Kai Gu, Ding Zhang, Shiqing Chen, Fengcai Wen, Hua Zhong, Baohui Han, Xueyan Zhang

**Affiliations:** ^1^ Department of Pulmonary, Shanghai Chest Hospital, Shanghai Jiao Tong University, Shanghai, China; ^2^ Medical Regulatory Affairs, Roche Diagnostics (Shanghai) Ltd., Shanghai, China; ^3^ The Medical Department, 3D Medicines Inc., Shanghai, China

**Keywords:** DDR, ctDNA = circulating tumor DNA, atezolizumab, predictive biomarker, non-small cell lung cancer

## Abstract

The presence of comutations (co-mut+) in DNA damage response and repair (DDR) pathways was associated with improved survival for immune checkpoint inhibitor (ICI) therapy in non-small cell lung cancer (NSCLC). However, it remains unknown whether co-mut+ status could be a predictive biomarker for immunotherapy. We aimed to explore the predictive role of co-mut+ status in the efficacy of ICIs. A total of 853 NSCLC patients from OAK and POPLAR trials were included in the analyses for the relationship between co-mut status and clinical outcomes with atezolizumab treatment. In co-mut+ NSCLC patients, significantly prolonged progression-free survival (PFS) (*p* = 0.004) and overall survival (OS) (*p* < 0.001) were observed in atezolizumab over docetaxel. The interaction between co-mut status and treatment was significant for PFS (*p* for interaction = 0.010) and OS (*p* for interaction = 0.017). In patients with negative or low programmed death receptor-ligand 1 expression, co-mut+ status still predicted improved clinical outcomes from atezolizumab therapy. These findings suggested that co-mut status may be a promising predictor of ICI therapy in NSCLC.

## Introduction

Lung cancer is the leading cause of cancer-related mortality worldwide with non-small cell lung cancer (NSCLC) being the most common type (1, 2). Recently, immune checkpoint inhibitors (ICIs) of programmed death receptor 1 (PD-1) and its ligand PD-L1 have improved outcomes in NSCLC patients (3, 4). However, due to the limited number of NSCLC patients benefiting from ICIs, there is an urgent need for robust predictor that can identify patients who are more likely to response to anti-PD-(L)1 immunotherapy.

Tumor mutational burden (TMB) is defined as the total number of tumor somatic mutations (5). Previous study also reported that higher TMB was correlated with improved response to immunotherapy and prolonged progression-free survival, regardless of PD-L1 expression (6). Additionally, the result of KEYNOTE-158 involving patients with previously treated, advanced solid tumors demonstrated a similar relationship between higher TMB (≥10 mutations/Mb) and pembrolizumab monotherapy (7). However, it is not appropriate to use a certain TMB cutoff in different tumors due to a wide distribution of burdens in various cancers (8). Our previous study revealed a non-linear association between blood-based tumor mutational burden (bTMB) and clinical outcome among NSCLC patients treated with atezolizumab (9). In consequence, the clinical utility of TMB or bTMB as a predictor of response to ICIs is limited by lack of a valid cutoff.

DNA damage response and repair (DDR) pathways counteract exogenous and endogenous sources of DNA damage and are essential for genomic integrity (10). Defective DDR pathways can promote cancer development (11). Previous research suggested that TMB was higher in patients with more DDR gene mutations in NSCLC patients (12). Furthermore, emerging evidence shows that various mutations in DDR pathways are correlated with clinical benefit from ICIs treatment (13–15).

Comutations (co-mut+) in the homologous recombination repair (HRR)–mismatch repair (MMR) and HRR–base excision repair (BER) pathways were found to be associated with high tumor immunogenicity and could identify patients that may potentially benefit from ICI therapy (16). However, without a control group, it remains unknown whether DDR comutations status could serve as a predictive biomarker in the ICI treatment setting. Furthermore, the function of mutations in DDR pathways has not been annotated in their study. In addition, owing to the challenge of obtaining sufficient tissue from patients with advanced NSCLC, blood-based detection for DDR mutations is an attractive surrogate for tissue-based test.

Thus, we conducted a study including patients from OAK and POPLAR studies, who received blood-based FoundationOne next-generation sequencing (NGS) testing. In this study, nonsense mutations and splice site alterations of DDR genes were considered deleterious. The deleterious status of missense mutations was determined by manual review in COSMIC and OncoKB databases. Co-mut+ were defined as deleterious alterations in two or more DDR pathways or deleterious alteration(s) in one DDR pathway plus missense mutation(s) of unknown significance in other DDR pathways. We hypothesized that co-mut+ status was associated with higher bTMB and may predict clinical benefits from atezolizumab treatment among NSCLC patients.

## Results

### Patient Characteristics

**Figure 1:**
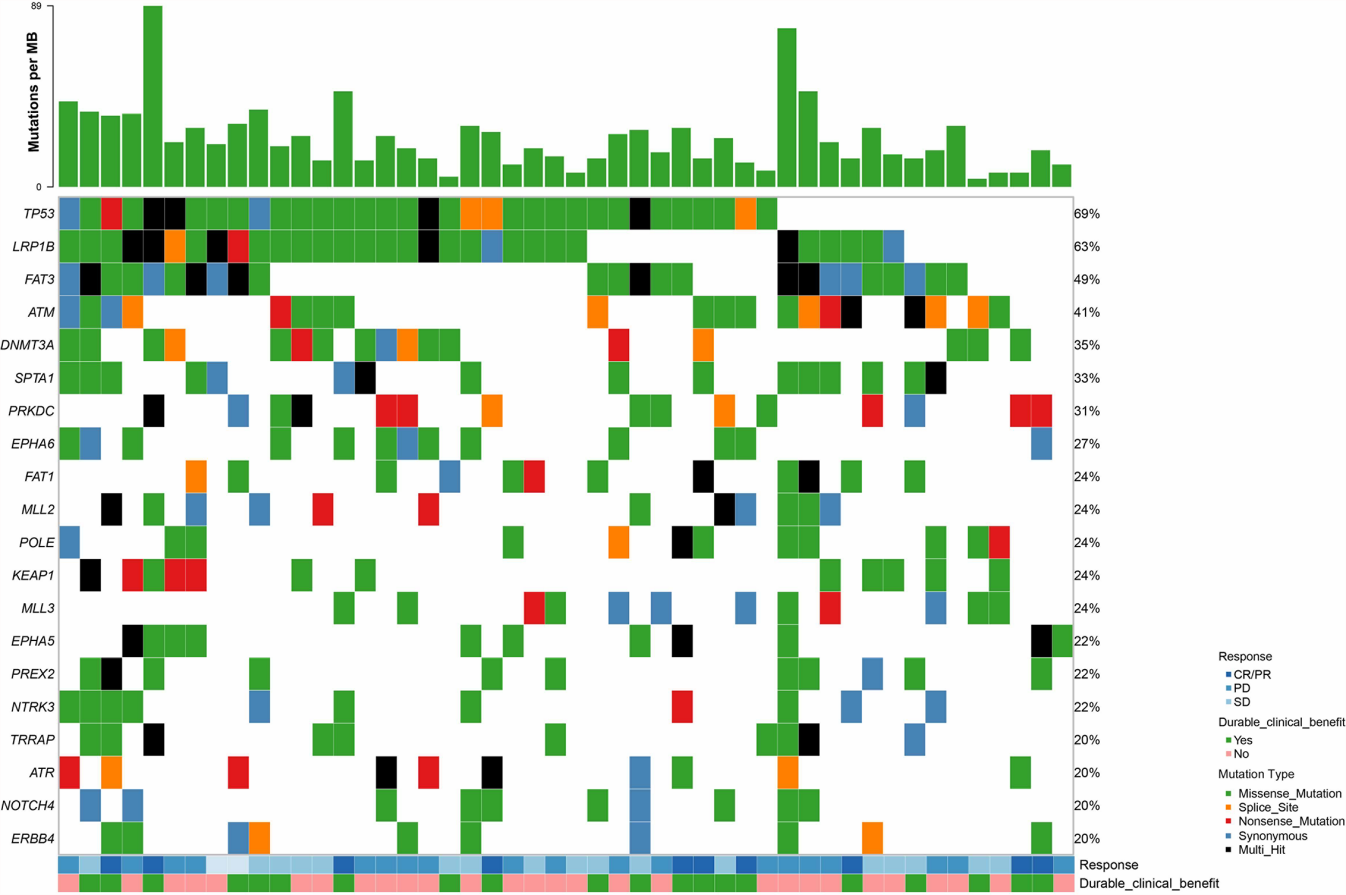
Patients’ mutation status and clinical data in co-mut+ group. bTMB, blood-based tumor mutational burden; CR, complete response; PR, partial response; PD, progressive disease; SD, stable disease.

A total of 853 patients were included in our analysis. Among them, there were 429 patients (50.3%) who received atezolizumab and the remaining 424 patients (49.7%) were treated with docetaxel. Co-mut+ status was found in 49 patients, accounting for 5.7% of all the patients. As is shown in **Table 1**, the demographic and clinical characteristics of the patients were well-balanced between co-mut+ and co-mut− groups except for baseline sum of the longest diameter (SLD), metastatic site, and bTMB. Co-mut+ patients had significantly larger baseline SLD (*p* = 0.006), more metastatic sites (*p* = 0.026), and higher bTMB (*p* < 0.001). The patients’ mutation status and clinical data are displayed in **Figure 1** and **Supplementary Figure 3**.

**Table 1:** Characteristics of patients with co-mut+ or co-mut− from OAK and POPLAR cohort.

Characteristics	Co-mut+ (*n* = 49)	Co-mut− (*n* = 804)	*p*
Age (years), median (IQR)	62.0 (57.0–68.0)	63.0 (57.0–70.0)	0.652
Race (%)			
White	34 (69.4)	582 (72.4)	0.649
Non-white	15 (30.6)	222 (27.6)	
Gender (% female)	16 (32.7)	310 (38.6)	0.409
Smoking status (%)			
Never	37 (75.5)	678 (84.3)	0.104
Former + Current	12 (24.5)	126 (15.7)	
ECOG PS (%)			
0	14 (28.6)	270 (33.6)	0.470
1	35 (71.4)	534 (66.4)	
Histology (%)			
Non-squamous	34 (69.4)	564 (70.1)	0.910
Squamous	15 (30.6)	240 (29.9)	
Line of therapy (%)			
Second	37 (75.5)	590 (73.4)	0.743
Third	12 (24.5)	214 (26.6)	
PD-L1 expression*			
TC0–2 or IC0–2	27 (81.8)	504 (82.8)	0.889
TC3 or IC3	6 (18.2)	105 (17.2)	
Treatment			
Atezolizumab	30 (61.2)	399 (49.6)	0.115
Docetaxel	19 (38.8)	405 (50.4)	
Baseline SLD (mm), median (IQR)	92.0 (57.0–144.0)	72.0 (45.0–105.0)	0.006
Metastatic site, median (IQR)	3.0 (2.5–4.0)	3.0 (2.0–4.0)	0.026
bTMB (mutation/Mb), median (IQR)	20.0 (13.5–29.5)	7.0 (3.0–15.0)	<0.001

ECOG, Eastern Cooperative Oncology Group; PS, performance status; IQR, interquartile range; SLD, sum of the longest diameter; PD-L1, programmed death-ligand 1; TC, tumor cell; IC, tumor infiltrating immune cells; bTMB, blood tumor mutation burden.

*n = 33 for the co-mut+ group and n = 609 for the co-mut− group.

Statistical significance was defined as two-sided p < 0.05.

PD-L1 expression was scored according to percentage of PD-L1 expressing tumor cells (TC3 ≥ 50%, TC2 ≥ 5% and <50%, TC1 ≥ 1% and <5%, and TC0<1%) and tumor-infiltrating immune cells (IC 3 ≥10%, IC2 ≥ 5% and <10%, IC1 ≥ 1% and <5%, and IC0<1%).

### Co-mut+ Status Predicted Higher Response to Atezolizumab

Co-mut+ status was significantly associated with increased bTMB (*p* < 0.001; **Supplementary Figure 1**). In the atezolizumab treatment group, proportion of durable clinical benefit (DCB) in co-mut+ patients was almost twice as that in co-mut− patients. Objective response rate (ORR) was 11.9% higher in co-mut+ patients than in co-mut− patients. In contrast, co-mut− patients exhibited higher DCB and ORR when they received docetaxel rather than atezolizumab (**Supplementary Figure 2**).

### Co-mut+ Status Predicted Favorable Clinical Outcome in Patients Treated With Atezolizumab

**Figure 2:**
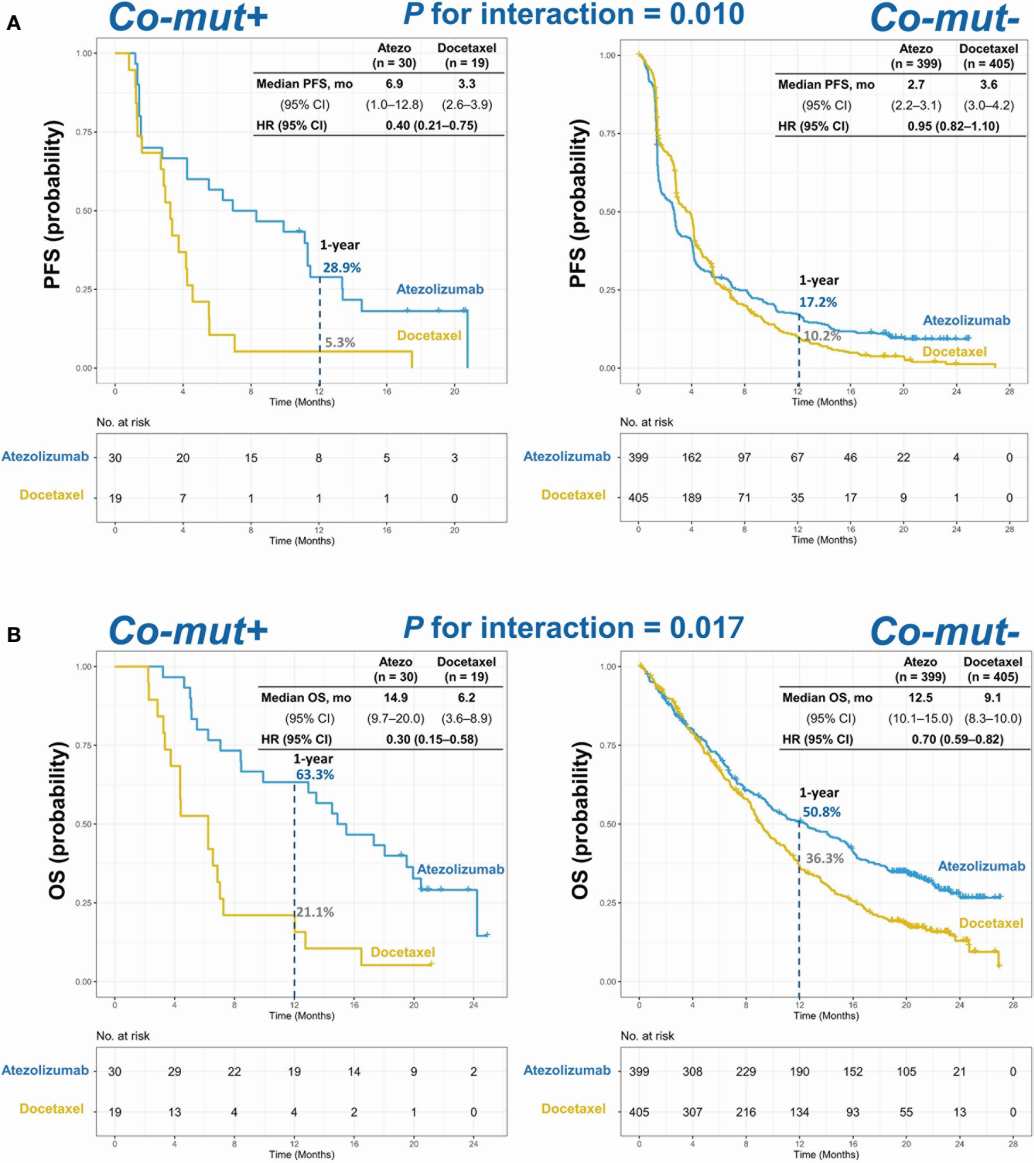
Kaplan–Meier estimates of **(A)** PFS and **(B)** OS in co-mut+ and co-mut− patients in atezolizumab and docetaxel treatment group. PFS, progression-free survival; OS, overall survival.

In co-mut+ patients, the atezolizumab treatment group presented with significantly longer progression-free survival (PFS) than did the docetaxel treatment group (adjusted hazard ratio (HR), 0.40; 95% CI, 0.21–0.75; *p* = 0.004], while no such correlation was observed in co-mut− patients [adjusted HR, 0.95; 95% CI, 0.82–1.10; *p* = 0.495). The interaction between co-mut status and treatment was significant for PFS (*p* for interaction = 0.010; **Figure 2A**). Additionally, co-mut+ patients treated with atezolizumab had a significantly prolonged overall survival (OS) compared with those treated with docetaxel (adjusted HR, 0.30; 95% CI, 0.15–0.58; *p* < 0.001). Despite longer OS in co-mut− patients in the atezolizumab group as compared with the docetaxel group due to the efficacy of the PD-L1 inhibitor (adjusted HR, 0.70; 95% CI, 0.59–0.82; *p* < 0.001), the *p* for interaction between comutations and treatment was also significant for OS (*p* for interaction = 0.017; **Figure 2B**).

### Co-mut+ Status Predict Improved Outcomes in Patients With Negative or Low PD-L1 Expression

We further investigated the relationship between co-mut status and the efficacy of atezolizumab and docetaxel in patients with negative or low PD-L1 expression. In this subgroup, interaction between treatment and co-mut status was still significant for OS (*p* for interaction = 0.010) and PFS (*p* for interaction = 0.036; **Supplementary Table 1**).

## Discussion

In this study, we found that co-mut+ status could predict improved clinical outcome with atezolizumab over docetaxel. Additionally, such predictive value was also observed in NSCLC patients with negative or low PD-L1 expression. Therefore, the presence of comutations in DDR pathways may be a predictive biomarker for ICI therapy.

The result of this study indicated that defective DDR pathways were associated with objective response with atezolizumab monotherapy. This might be related to elevated mutation burden and activation of the stimulator of interferon genes (STING) pathway. Firstly, defects in DDR pathways contribute to the accumulation of genomic errors and generation of tumor-specific neoantigens, resulting in increased tumor immunogenicity. Correspondingly, higher bTMB was observed in co-mut+ patients in our research. Secondly, DDR deficiency can result in increased cytosolic DNA, which can be sensed by the cyclic GMP-AMP synthase (cGAS) and lead to activation of innate immune response *via* STING pathway. Consequently, elevated PD-L1 expression as well as T-cell recruitment by proinflammatory cytokine CXCL10 and CCL5 occur in the tumor microenvironment (17, 18).

The association between co-mut+ status and objective response to ICIs was still significant in patients with negative or low PD-L1 expression in this study. Currently, PD-L1 expression has been considered as a standard predictive biomarker of PD-(L)1 inhibitor response (19). However, it is an imperfect predictor with drawbacks of heterogeneity of expression and test platform uniformities. Furthermore, objective response was also observed in tumors with <1% PD-L1 expression when first-line PD-L1 inhibitor was used for advanced NSCLC (20). Notably, our study showed that co-mut+ patients with negative or low PD-L1 expression could also derive benefit from atezolizumab compared with docetaxel. As a result, co-mut+ status may be a potential predictive biomarker for ICIs treatment even in NSCLC patients with <1% PD-L1 expression.

Our research demonstrated that co-mut+ status was a potential promising predictive biomarker for atezolizumab treatment in NSCLC. TMB is also an attractive predictor of response to ICIs. Still, lack of a validated cutoff value limited the utilization of TMB in clinical work. By contrast, it is more convenient to apply co-mut status as a predictive biomarker owing to its binary nature. Although MMR deficiency has been approved as an indication of application of pembrolizumab irrespective of the origin of tumors by the Food and Drug Administration (FDA) (21), the frequency of mismatch-repair-deficient (dMMR) was reported to be less than 2% in lung squamous cell carcinoma and lung adenocarcinoma (22). Hence, co-mut status is a more feasible and promising predictive biomarker for ICI therapy as compared with TMB or dMMR.

Previous study has demonstrated that deleterious DDR mutations were associated with better clinical outcomes with ICIs in NSCLC (14). However, only six patients had more than one deleterious altered DDR gene and no such correlation was observed in our study (results not shown). The possible reason was that only 29 DDR genes were included in the FoundationOne NGS panel, whereas 53 DDR genes were in their study. Therefore, we recommend that more DDR genes should be included in the NGS panel. In addition, the potential association between mutated *POLE* and clinical benefit of ICIs has been revealed in NSCLC (23). However, the low incidence of the specific gene mutation in NSCLC may limit its clinical utility as a predictive biomarker. Our definition of co-mut+ status was not limited to a certain gene and might be a more feasible predictor of ICIs treatment.

DDR mutation profiling was determined using whole-exome or whole-genome sequencing from tissue sample in the previous study (16). However, for most advanced NSCLC patients, the availability of adequate tissue for molecular tests has become increasingly limited. In our study, co-mut status detection using circulating tumor DNA was associated with improved survival after atezolizumab treatment. Therefore, blood-based comutations in DDR pathways could serve as a more convenient and less invasive approach to guide ICI therapy than a tumor biopsy.

However, there are still some limitations. Firstly, this study has inevitable bias due to its retrospective nature. Secondly, there is only one atezolizumab-treated cohort in our research and our results need to be confirmed in larger cohorts of patients. Thirdly, most other DDR genes were not included in the panel used in this study. Fourthly, annotation of mutations was based on manual review in COSMIC and OncoKB databases. However, mutations are annotated theoretically and need to be further confirmed.

In conclusion, co-mut+ status was related to enhanced bTMB and predicted the clinical benefits of atezolizumab in NSCLC patients, which should be validated by further studies.

## Method

### Data Source

**Figure 3:**
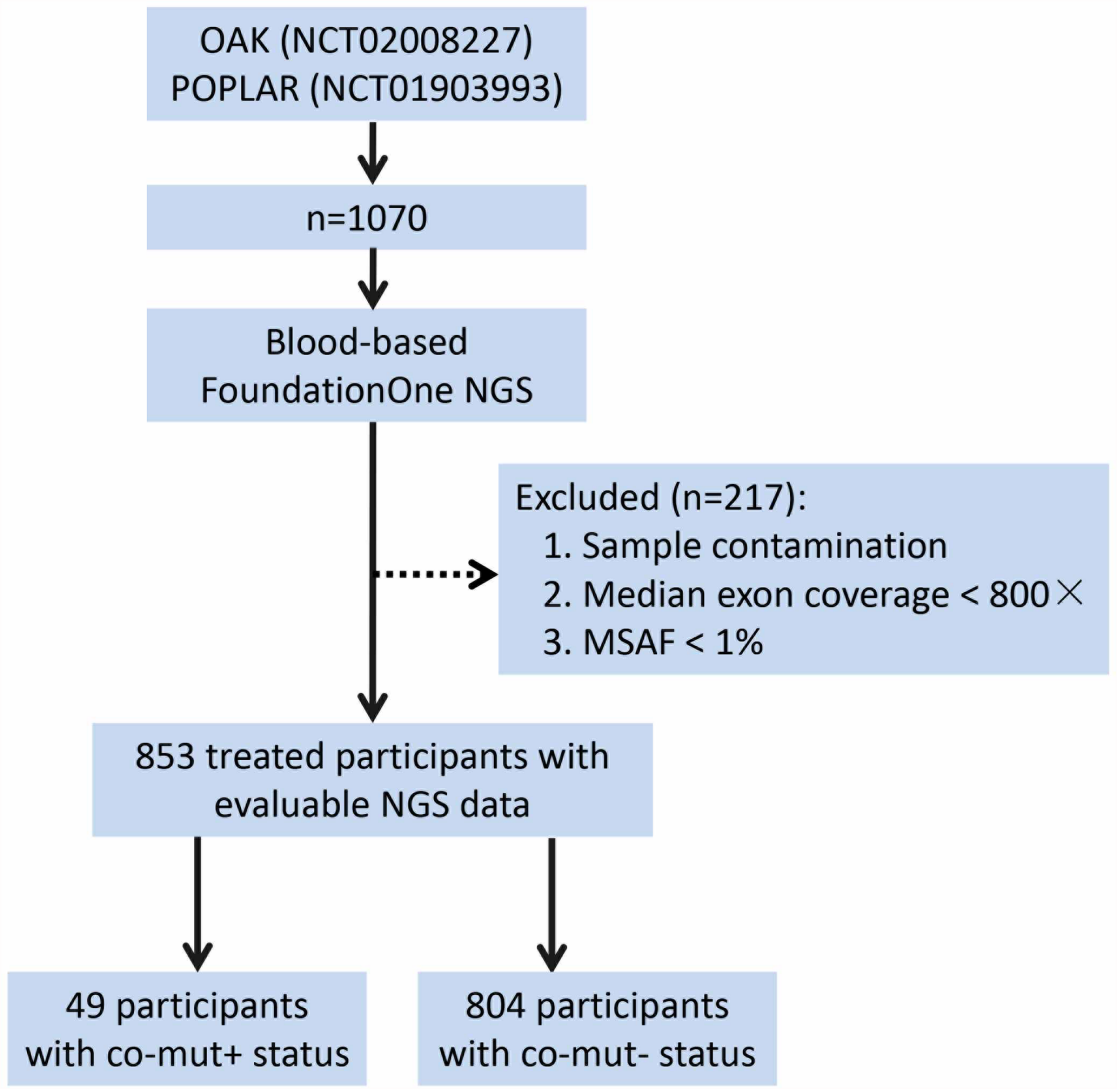
Process of patient selection. NGS, next-generation sequencing; MSAF, maximum somatic allele frequency.

The design and methods of OAK and POPLAR trials were reported previously (24, 25). Patients in both studies received cytotoxic chemotherapy and then were randomly assigned to treatment with atezolizumab (1,200 mg fixed dose every 3 weeks) or docetaxel (75 mg/m (2) every 3 weeks). Two studies were performed in accordance with the guidelines for Good Clinical Practice and the Declaration of Helsinki, and written informed consent was received from all patients. As the aim of this study is to analyze the role of co-mut status in atezolizumab treatment, 853 patients with evaluable NGS data were included (**Figure 3**). This study was conducted without institutional review board approval, as all the data were extracted from published papers (26).

### Genetic Analysis

FoundationOne NGS testing was performed for the 853 patients to determine genetic alterations (**Supplementary Table 2**) (27, 28). The detection of bTMB has been described by Gandara et al. (26). To calculate bTMB score, all single-nucleotide variants with allele frequencies of ≥0.5% were counted and germline mutations were filtered out by comparing against the dbSNP and ExAC databases.

### Definition of Comutations in DDR Pathways

Twenty-nine DDR genes in seven categories were detected in this study (**Supplementary Table 2**). Nonsense and splice site mutations in DDR genes were considered deleterious. The identified missense mutations were manually reviewed in COSMIC, OncoKB, and Foundation Medicine proprietary databases and reported pathogenic mutations were classified as deleterious. Co-mut+ status would be confirmed if one of the following criteria was fulfilled: (1) deleterious alterations in two or more DDR pathways; (2) deleterious alteration(s) in one DDR pathway plus missense mutation(s) of unknown significance in other DDR pathway(s).

### Outcomes

OS, PFS, DCB, and ORR were assessed. OS was defined as the time from treatment initial until death from any cause. PFS was defined as the time interval from the date of randomization to the date of first documented disease progression or death resulting from any cause. DCB was defined as PFS lasting ≥6 months. ORR was defined as the proportion of patients with a confirmed complete response (CR) or a partial response (PR) by RECIST v1.1.

### Statistical Analysis

*χ*² test was used to examine the differences in baseline and clinical characteristics between co-mut+ and co-mut− group. The Kaplan–Meier method was used to calculate median OS and PFS. HRs were calculated from Cox proportional‐hazard models. The *p*-value for interaction was calculated from the proportional Cox model including the variables of therapy, the co-mut status, and treatment by the co-mut status interaction. Mann–Whitney *U* test was used to examine the difference of bTMB between co-mut+ and co-mut− groups. Statistical significance was defined as two-sided *p* < 0.05. All analyses were conducted with R, version 3.6.1 (R Project for Statistical Computing) and SPSS version 24.0 (IBM, Armonk, NY).

## Data Availability Statement

The data of the OAK and POPLAR trials included in this analysis were provided online by F. Hoffmann–La Roche (https://www.nature.com/articles/s41591-018-0134-3#Sec19). Datasets analyzed during our study are available from the corresponding authors on reasonable request. Additionally, the data used in the current study have been analyzed in our previous studies (https://doi.org/10.1080/2162402X.2020.1731072) (https://doi.org/10.6004/jnccn.2019.7383) (https://doi.org/10.1002/ijc.32717).

## Author Contributions

Conceptualization: AX and WN. Literature search: AX, YZ, and FW. Figures: CL and KG. Data collection and analysis: DZ, SC, and HZ. Writing—original draft: AX and WN. Writing—review and editing: BH and XZ. All authors contributed to the article and approved the submitted version.

## Funding

This study was supported in part by the Shanghai Chest Hospital Project of Collaborative Innovation (Grant Number: YJXT20190102) and the National Natural Science Foundation of China (Grant Number: 81601988).

## Conflict of Interest

Author KG is employed by Roche Diagnostics (Shanghai) Ltd. Authors DZ, SC, and FW are employed by 3D Medicines Inc.

The remaining authors declare that the research was conducted in the absence of any commercial or financial relationships that could be construed as a potential conflict of interest.

## Publisher’s Note

All claims expressed in this article are solely those of the authors and do not necessarily represent those of their affiliated organizations, or those of the publisher, the editors and the reviewers. Any product that may be evaluated in this article, or claim that may be made by its manufacturer, is not guaranteed or endorsed by the publisher.
