# Supplementary material for: Comutations in DDR Pathways Predict Atezolizumab Response in Non-Small Cell Lung Cancer Patients
Source: Front Immunol. 2021 Sep 24;12:708558. doi: 10.3389/fimmu.2021.708558 (PMC8499805; doi:10.3389/fimmu.2021.708558)
Supplement: Supplementary file 1 [file DataSheet_1.docx]

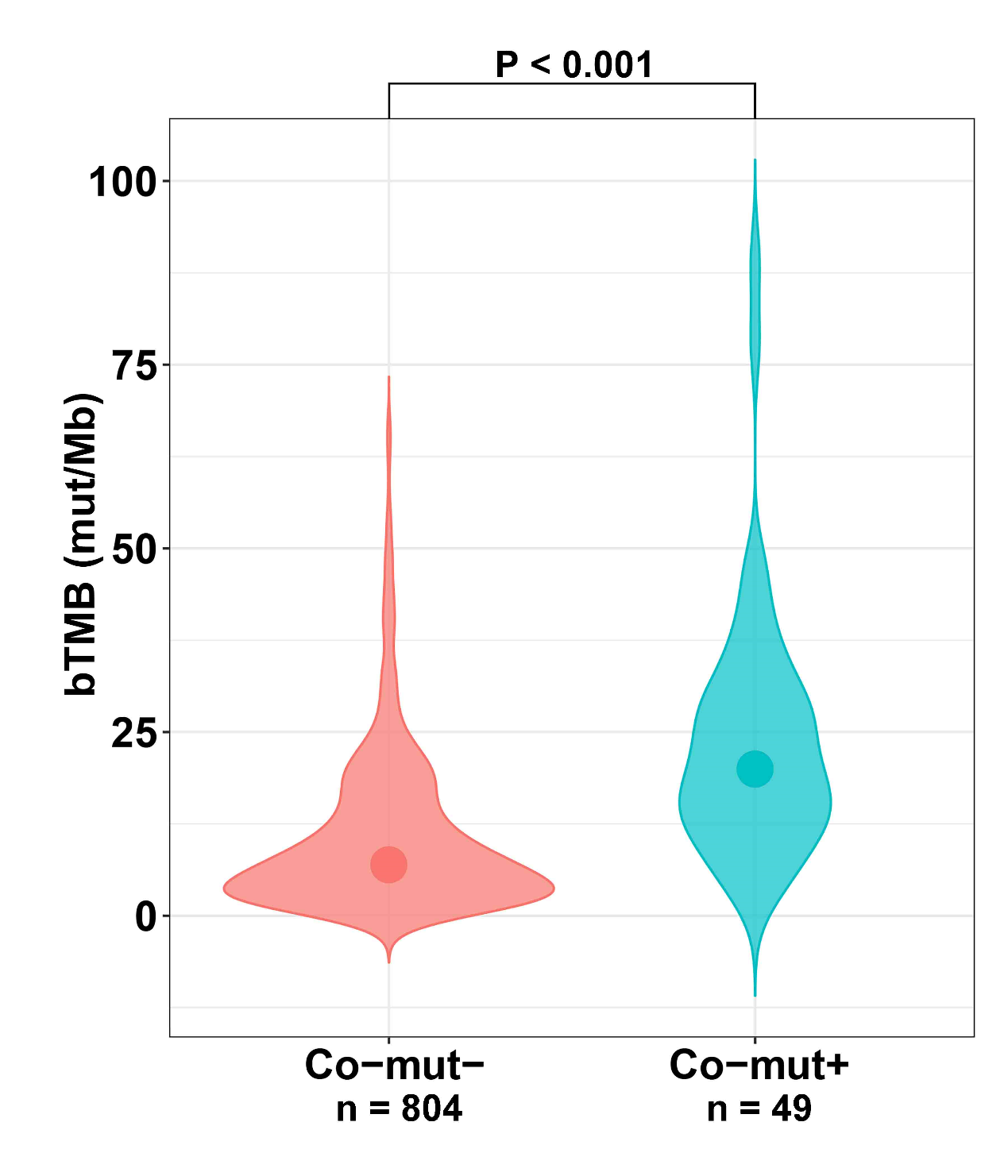


**Supplementary Figure 1.** bTMB in co-mut+ and co-mut- patients.

Abbreviations: bTMB, blood-based tumor mutational burden.


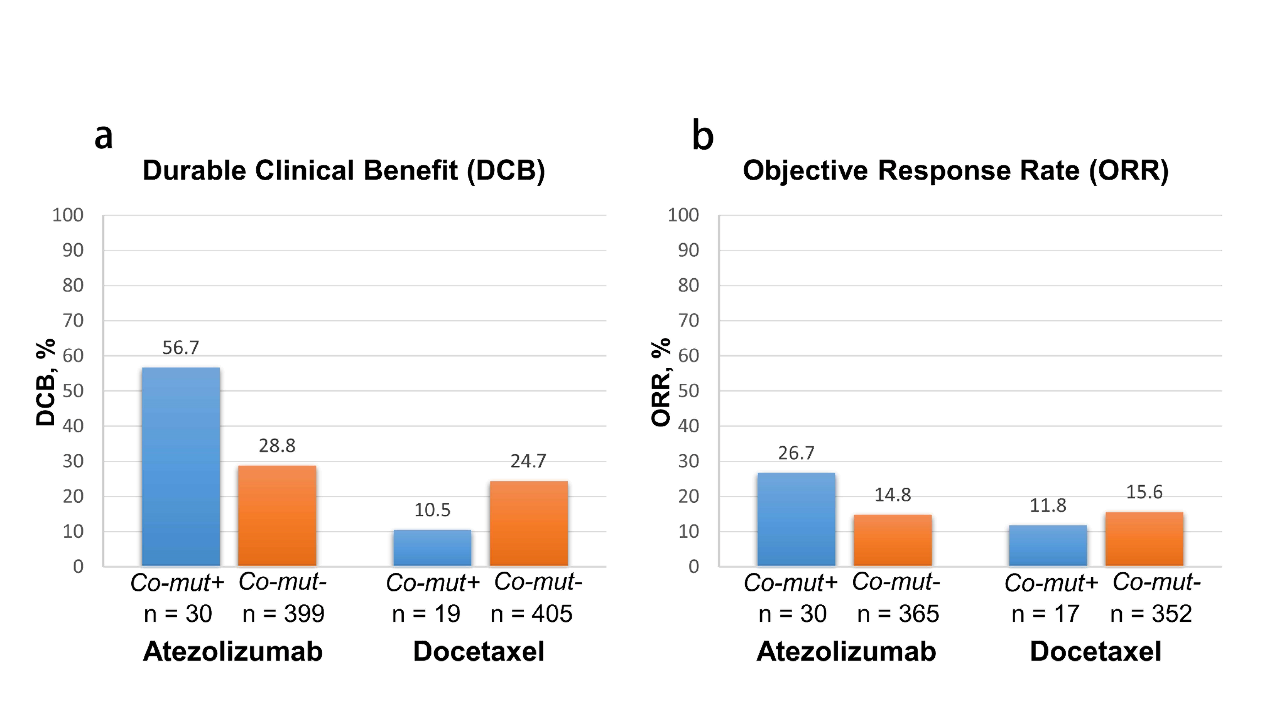


**Supplementary Figure 2.** Comparison of (a) durable clinical benefit and (b) objective response rate between co-mut+ and co-mut- patients in atezolizumab and docetaxel treatment group.


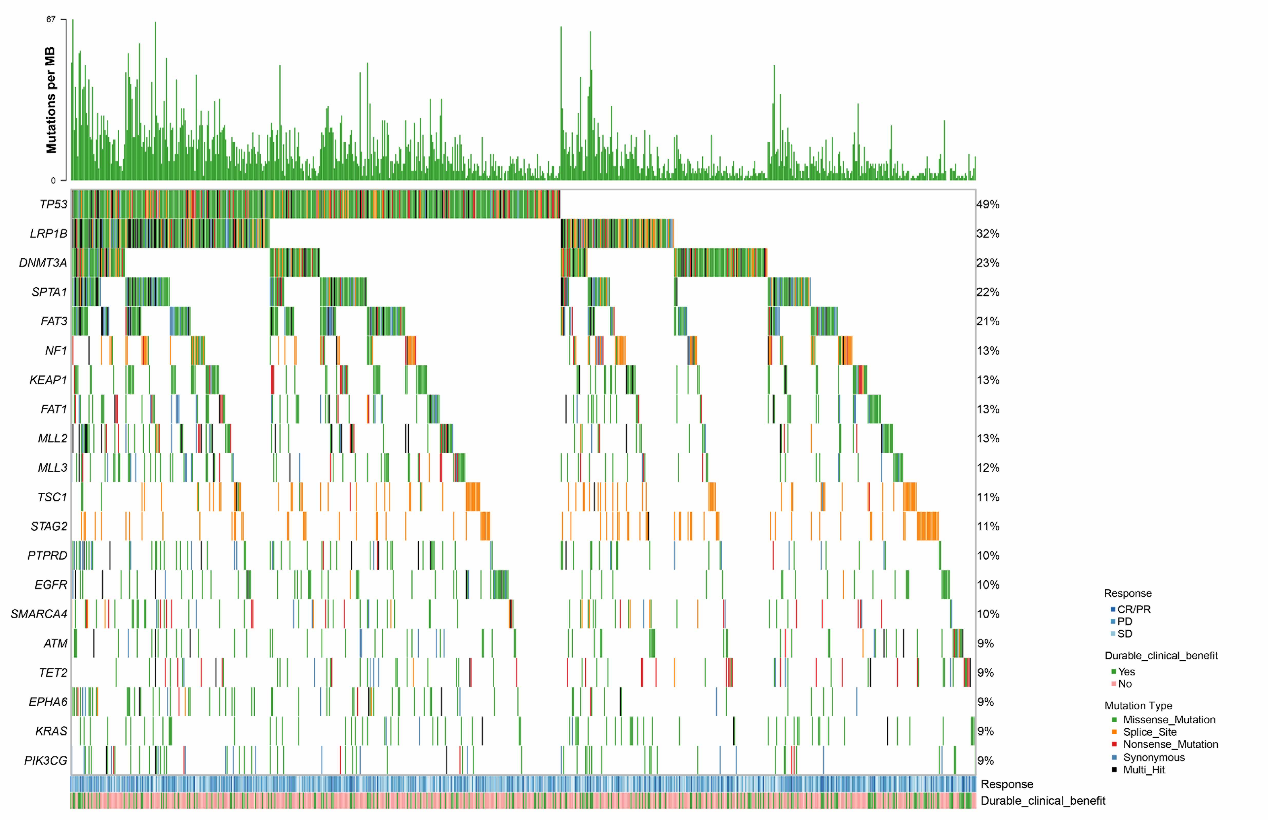


**Supplementary Figure 3.** Patients’ mutation status and clinical data in co-mut- group.

Abbreviations: bTMB, blood-based tumor mutational burden; CR, complete response; PR, partial response; PD, progressive disease; SD, stable disease.

**Supplementary Table 1.** Treatment interaction with comutations in DDR pathways for overall survival and progression-free survival in patients with negative or low PD-L1 expression

|  | OS | | | PFS | | |
| --- | --- | --- | --- | --- | --- | --- |
|  | HR | 95% CI | *P* for interaction | HR | 95% CI | *P* for interaction |
| Co-mut+ | 0.173 | 0.058-0.519 | 0.010 | 0.363 | 0.140-0.940 | 0.036 |
| Co-mut- | 0.756 | 0.616-0.929 |  | 1.026 | 0.852-1.236 |  |

Abbreviations: OS, overall survival; PFS, progression-free survival; HR, hazard ratio; CI, confidence interval.

Negative or low PD-L1 expression was defined as TC0-2 or IC0-2. PD-L1 expression was scored according to percentage of PD-L1 expressing tumor cells (TC3≥50%, TC2≥5% and ＜50%, TC1≥1% and ＜5%, and TC0＜1%) and tumor-infiltrating immune cells (IC3≥10%, IC2≥5% and ＜10%, IC1≥1% and ＜5%, and IC0＜1%).

**Supplementary Table 2.** Genes included in the blood-based FoundationOne NGS panel.
